# Sperm Proteome Analysis and Identification of Fertility-Associated Biomarkers in Unexplained Male Infertility

**DOI:** 10.3390/genes10070522

**Published:** 2019-07-11

**Authors:** Manesh Kumar Panner Selvam, Ashok Agarwal, Peter Natesan Pushparaj, Saradha Baskaran, Hocine Bendou

**Affiliations:** 1 American Center for Reproductive Medicine, Cleveland Clinic, Cleveland, OH 44195, USA; 2 Center of Excellence in Genomic Medicine Research, King Abdulaziz University, Jeddah 21589, Saudi Arabia; 3 Department of Medical Laboratory Technology, Faculty of Applied Medical Sciences, King Abdulaziz University, Jeddah 21589, Saudi Arabia; 4 South African National Bioinformatics Institute (SANBI), SA Medical Research Council Bioinformatics Unit, University of the Western Cape, Private Bag X17, Bellville, Cape Town 7535, South Africa

**Keywords:** male infertility, normozoospermic infertile men, unexplained male infertility, sperm proteomics, biomarkers

## Abstract

Up to 30% of men with normal semen parameters suffer from infertility and the reason for this is unknown. Altered expression of sperm proteins may be a major cause of infertility in these men. Proteomic profiling was performed on pooled semen samples from eight normozoospermic fertile men and nine normozoospermic infertile men using LC-MS/MS. Furthermore, key differentially expressed proteins (DEPs) related to the fertilization process were selected for validation using Western blotting. A total of 1139 and 1095 proteins were identified in normozoospermic fertile and infertile men, respectively. Of these, 162 proteins were identified as DEPs. The canonical pathway related to free radical scavenging was enriched with upregulated DEPs in normozoospermic infertile men. The proteins associated with reproductive system development and function, and the ubiquitination pathway were underexpressed in normozoospermic infertile men. Western blot analysis revealed the overexpression of annexin A2 (ANXA2) (2.03 fold change; *P* = 0.0243), and underexpression of sperm surface protein Sp17 (SPA17) (0.37 fold change; *P* = 0.0205) and serine protease inhibitor (SERPINA5) (0.32 fold change; *P* = 0.0073) in men with unexplained male infertility (UMI). The global proteomic profile of normozoospermic infertile men is different from that of normozoospermic fertile men. Our data suggests that SPA17, ANXA2, and SERPINA5 may potentially serve as non-invasive protein biomarkers associated with the fertilization process of the spermatozoa in UMI.

## 1. Introduction

Male infertility is a global problem that accounts for 50% of infertility cases [1]. Several conditions are associated with the incidence of male infertility. These include both congenital and acquired conditions such as varicocele, genital tract infection, hormonal disturbance, genetic defects, lifestyle changes, exposure to toxins, etc. [2]. Infertility in men is also reported due to unknown causes and is either termed idiopathic or unexplained male infertility (UMI). Idiopathic male infertility is associated with an abnormal semen parameter [3]. UMI is the inability of the male to establish pregnancy, despite having normal semen parameters without any known cause of infertility and the absence of a female infertility factor [4]. Laboratory evaluation of male infertility begins with conventional semen analysis, which includes key sperm parameters such as concentration, motility, morphology, and viability [5]. Abnormal semen parameters are considered an indicator of subfertility in men. However, up to 30% of men with normal semen parameters (normozoospermic) are diagnosed with UMI as the reason for infertility is unknown [3,6].

The subcellular factors associated with the fertilization failure of spermatozoa are not clearly explained with conventional semen analysis. Proteomics has emerged as an important tool to identify the underlying etiology in defective spermatozoa. High throughput techniques such as LC-MS/MS are able to effectively profile the proteins present in the spermatozoa [7]. A change in the expression of the sperm proteins may be a major cause of subfertility in normozoospermic men. The molecular mechanisms associated with sperm functions, such as motility, capacitation, an acrosomal reaction, and fertilization (sperm–oocyte interaction), are reported to be altered in the spermatozoa of infertile men with normozoospermic semen parameters [8]. The dysregulated proteins have been related to sexual reproduction, the metabolic process, cell growth and/or maintenance, protein metabolism, and protein transport [9]. In addition, the proteins involved in the chromatin assembly process have been shown to be defective in in vitro fertilization failure spermatozoa from normozoospermic infertile men [10,11].

Although earlier proteomic studies provided evidence of defective sperm proteins in UMI [8,12,13], the identification of proteins was limited by the 2D-electophoresis technique [14]. There is a chance of missing less abundant proteins expressed in the spermatozoa. Robust shot-gun proteomic techniques such as LC-MS/MS can detect the maximum number of peptides in a given sample. Recently, Azpiazu et al., used the LC-MS/MS platform to profile the sperm proteins in normozoospermic infertile men. However, only one protein serine/arginine-rich splicing factor kinase 1 (SRPK1) was validated using the Western blot technique [10]. This study was designed to (1) compare the quantitative sperm proteome of normozoospermic infertile men (UMI) with normozoospermic fertile men, and (2) validate the proteins associated with the fertilization process using the conventional Western blot technique.

## 2. Materials and Methods

### 2.1. Study Participants and Semen Analysis

This study was approved by the Institutional Review Board (IRB) of the Cleveland Clinic (#11-451). All participants signed an informed written consent form at the Andrology Center, Cleveland Clinic. The semen samples from healthy normozoospermic fertile men (n = 8) and normozoospermic infertile men/UMI subjects (n = 9) were used for proteomic analysis in compliance with the Minimum Information about a Proteomics Experiment (MIAPE) guidelines of the Human Proteome Organization’s Proteomics Standards Initiative (HUPO-PSI) for reporting proteomics studies [15]. Semen samples were collected after 2–3 days of sexual abstinence and were allowed to liquefy completely for 20–30 min at 37 °C. Semen volume, sperm motility, and concentration were evaluated according to the World Health Organization (WHO) 2010 guidelines [5]. Sperm parameters were considered normal when the semen volume was ≥1.5 mL, sperm concentration was ≥15 million/mL, total sperm motility was ≥40%, and normal sperm morphology was ≥4%, as per the WHO criteria [5]. Semen samples from all the subjects were cryopreserved in TEST-yolk buffer (TYB; Irvine Scientific, Santa Ana, CA, USA) using slow freezing protocol [16].

### 2.2. Inclusion and Exclusion Criteria

All the normozoospermic healthy fertile men included in this study had initiated pregnancy or fathered a child in the last two years. However, the normozoospermic infertile men had failed to establish pregnancy after two years of unprotected intercourse, with their female partner having normal reproductive health. All participants included in the study were nonsmokers, non-alcoholic, and had a normal body mass index.

Men with leukocytospermia (Endtz positive), varicocele, azoospermia, and oligozoospermia (<10^6^ sperm mL^−1^) were excluded from the study. Patients with a recurring fever prior to 90 days of semen analysis were excluded. Infertile men with genetic defects and a reproductive tract infection diagnosed by andrological examination were not included in the present study. All the study participants with a history of erectile dysfunction, systemic illness, inflammation of the reproductive tract (orchitis, epididymitis, urethritis, and testicular atrophy), and sexually transmitted diseases were also excluded.

### 2.3. Sperm Protein Extraction and Quantification

Samples were thawed at 37 °C for 20 min and centrifuged at 4000× *g* for 10 min to isolate spermatozoa. The sperm pellet was washed four times with phosphate buffered saline (PBS; Irvine Scientific, Santa Ana, CA, USA) and centrifuged at 4000× *g* for 10 min, at 4 °C. Radio-immunoprecipitation assay (RIPA; Sigma-Aldrich, St. Louis, MO, USA) buffer supplemented with Protease Inhibitor Cocktail, cOmplete™ ULTRA Tablets, EDTA-free (Roche, Mannheim, Germany) was added to the sperm pellet (100 µL RIPA/10^6^ sperm) and left overnight at 4 °C for cell lysis. Samples were centrifuged at 10,000× *g* for 30 min, at 4 °C, and the supernatant was transferred to a new centrifuge tube. Protein quantification in the fractions was performed using the Pierce BCA Protein Assay kit (Thermo Fisher Scientific, Waltham, MA, USA), according to the manufacturer’s instructions.

### 2.4. Liquid Chromatography-Tandem Mass Spectrometry

Pooled samples from eight normozoospermic fertile men and nine from normozoospermic infertile men were used for global proteomic analysis by LC-MS/MS. The samples in each pool were mixed with SDS-PAGE buffer and separated on a 1D gel and run in triplicate. For protein digestion, the bands were cut to minimize excess polyacrylamide, and divided into a number of smaller pieces. After washing the gel pieces with water and dehydrating them in acetonitrile, the bands were reduced with dithiothreitol and alkylated with iodoacetamide. Subsequently, all bands were digested in-gel using trypsin, by adding five μL of 10 ng/μL trypsin to 50 mM ammonium bicarbonate and incubating the sample overnight at room temperature to achieve complete digestion. The peptides formed were extracted from the polyacrylamide in two aliquots of 30 μL 50% acetonitrile with 5% formic acid. These extracts were combined and evaporated to <10 μL in Speedvac and then resuspended in 1% acetic acid to make up a final volume of ~30 μL for LC-MS analysis.

The LC-MS/MS system was a Finnigan LTQ-Orbitrap Elite hybrid mass spectrometer system. The HPLC was performed using a Dionex 15 cm × 75 μm id Acclaim Pepmap C18, 2 μm, 100 Å reversed phase capillary chromatography column, as described in our previous publication [17]. The data was analyzed using all collision-induced dissociation (CID) spectra collected in the experiment to search the human reference sequence databases (http://www.hprd.org/) with the search program Mascot and Sequest. Protein identification criteria were established at a >99% probability to achieve a false detection rate (FDR) <1%. These search results were then uploaded into the program Scaffold (Proteome Software Inc., Portland, OR, USA; version 4.0.6.1). The abundance of each protein in the pool was classified as very low, low, medium, or high, based on the number of spectral counts (SC). The normalized spectral abundance factor (NSAF) ratio was calculated to categorize the expression profile of differentially expressed proteins (DEPs) as underexpressed, overexpressed, or unique to one of the groups [17]. The error observed in the SC measurements was greater for less abundant proteins compared to more highly abundant proteins. Due to this, different filtering criteria were used to determine if proteins were differentially present based on the overall abundance. Different constraints for significance tests (*p* value) and/or fold change cutoffs (or the NSAF ratio) were applied to these four abundance categories, as shown below:

1. Very Low abundance: SC range 1.7–7; *p* ≤ 0.001 and NSAF ratio ≥2.5 for overexpressed, ≤0.4 for underexpressed proteins;
2. Low abundance: SC range 8–19; *p* ≤ 0.01 and NSAF ratio ≥2.5 for overexpressed, ≤0.4 for underexpressed proteins;
3. Medium abundance: SC range between 20 and 79; *p* ≤ 0.05 and NSAF ratio ≥2.0 for overexpressed, ≤0.5 for underexpressed proteins;
4. High abundance: SC >80; *p* ≤ 0.05 and NSAF ratio ≥1.5 for overexpressed, ≤0.67 for underexpressed proteins.

### 2.5. Bioinformatic Analysis

Ingenuity Pathway Analysis (IPA) software (Version: 48207413) (Qiagen, Hilden, Germany) was used to perform functional pathway analysis of the DEPs. IPA allows the evaluation of top canonical pathways, diseases and bio-functions, causal networks, and upstream regulators related to the DEPs.

### 2.6. Protein Selection and Validation by Western Blotting

To validate the global proteomic findings, the sperm proteins related to reproductive function were selected for validation by Western blotting (WB) in a different set of samples from normozoospermic fertile men and normozoospermic infertile men to maintain the biological variability. The criteria for the selection of DEPs for validation by WB included (1) proteins involved in reproductive system development and function, (2) proteins involved in the top canonical pathways, (3) proteins with a well-described function in the literature, and (4) proteins with a moderate or high abundance in any one of the experimental groups. Four proteins (ANXA2, PRDX2, SPA17, and SERPINA5) were selected for validation by WB in individual samples from normozoospermic fertile men (n = 10) and infertile men (n = 10). A total of 20 µg of protein per sample was loaded into a 4%–15% SDS–PAGE for 2 h at 90 V. The resolved protein bands were then transferred onto polyvinylidene difluoride (PVDF) membranes and were blocked for 90 min at room temperature, with a 5% non-fat milk solution prepared in tris-buffered saline tween-20. For each protein analysis, specific primary antibodies were incubated at 4 °C overnight (Table S1). Then, the membranes were incubated with the secondary antibody at room temperature for 1 h and finally reacted with enhanced chemiluminescence (ECL) reagent (GE Healthcare, Marlborough, MA, USA) for 5 min. Membranes were exposed to Chemi-Doc (ChemiDoc™ MP Imaging System, Bio-Rad, Hercules, CA, USA) to detect the chemiluminescence signals.

All the PVDF membranes used for protein identification were subjected to total protein staining. The membranes were briefly washed twice for 10 min in distilled water and stained with total colloidal gold protein stain (Bio-Rad) for 2 h at room temperature by gentle shaking. Stained membranes were washed twice with distilled water for 10 min, and the densitometry image was captured using the colorimetric mode on Chemi-Doc (ChemiDoc™ MP Imaging System, Bio-Rad).

### 2.7. Statistical Analysis

A moderated t-test was applied to establish the significance levels for DEPs. Data analysis was performed using MedCalc Statistical Software (version 17.8; MedCalc Software, Ostend, Belgium). After testing for a normal distribution using the Kolmogorov–Smirnov test, a Mann–Whitney test was carried out to compare the semen parameters of normozoospermic fertile men and normozoospermic infertile men, and a *P* value < 0.05 was considered significant. The same test was used to compare the expression levels of the proteins validated using the Western blot technique in both the groups.

## 3. Results

### 3.1. Semen Parameters

Semen parameters of the normozoospermic fertile and normozoospermic infertile men are presented in Tables S2 and S3. No significant difference (*p* > 0.05) was observed in the sperm concentration, motility, and morphology between the two groups.

### 3.2. Sperm Proteome of Normozoospermic Fertile and Normozoospermic Infertile Men

**Figure 1:**
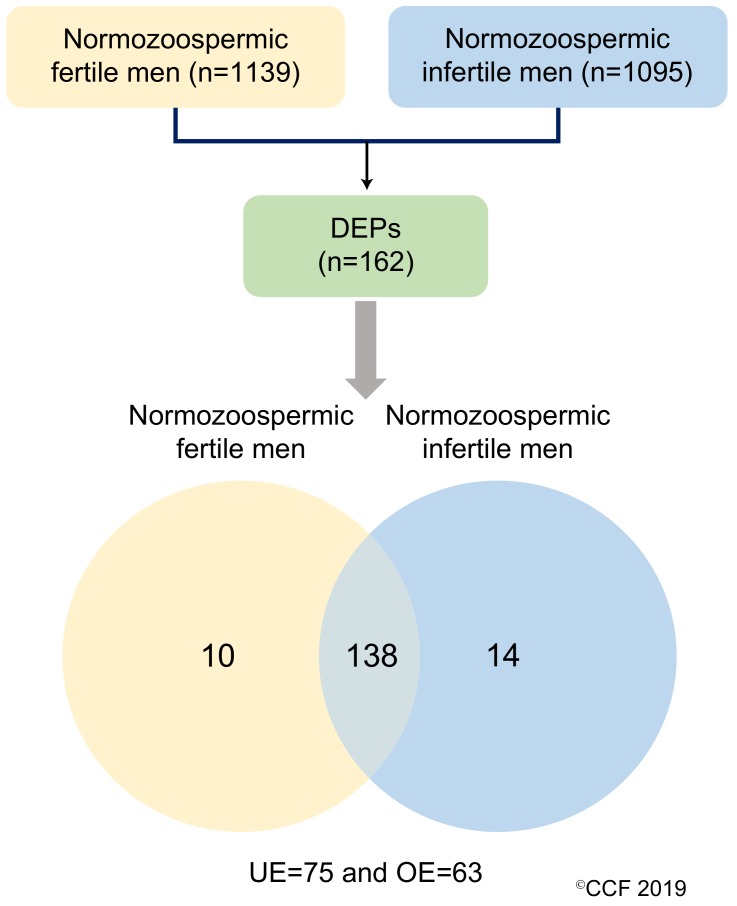
Differentially expressed proteins (DEPs) in normozoospermic fertile men and normozoospermic infertile men. UE: underexpressed and OE: overexpressed.

LC-MS/MS detected a total of 1139 and 1095 proteins in normozoospermic fertile men and normozoospermic infertile men, respectively. Based on the NSAF ratio and protein abundance, 162 were identified as DEPs (Table S4). The overexpressed and underexpressed DEPs, and unique proteins are shown in Figure 1.

### 3.3. Key Canonical Pathways Enriched in Normozoospermic Infertile Men

**Figure 2:**
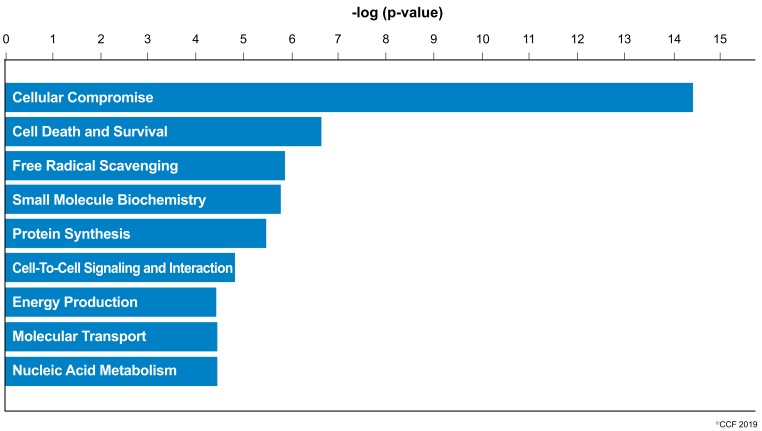
Molecular and cellular functions enriched in the normozoospermic infertile men.

**Figure 3:**
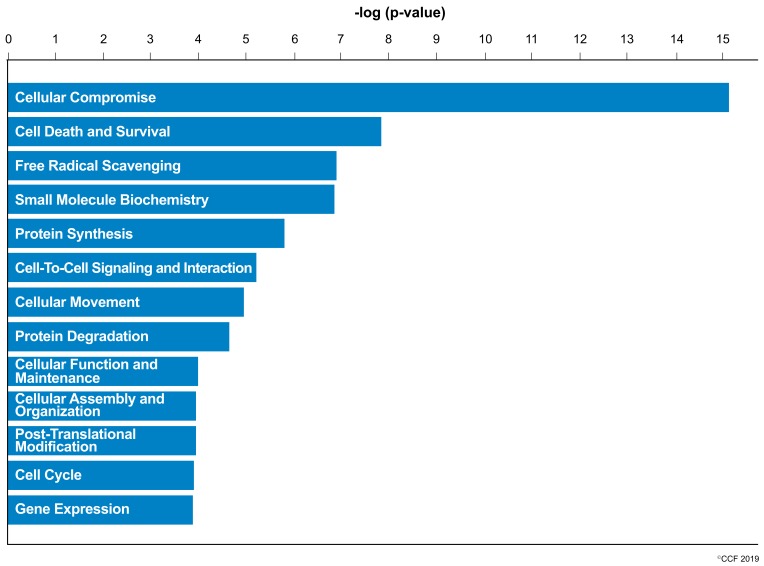
Key canonical pathways enriched in normozoospermic infertile men due to the involvement of overexpressed differentially expressed proteins.

IPA revealed cellular compromise as the top enriched canonical pathway, followed by cell death and survival, and free radical scavenging pathways. A list of all the canonical pathways associated with the expression of DEPs (both upregulated and downregulated proteins) in normozoospermic infertile men and normozoospermic fertile men are depicted in Figure 2. A comparative analysis of the DEPs in normozoospermic infertile men compared to upregulated DEPs in normozoospermic fertile men showed that the canonical pathway related to free radical scavenging was enriched with upregulated DEPs in normozoospermic infertile men (Figure 3).

An in-depth functional analysis of sperm proteins revealed that SPA17 and fibronectin (FN1) associated with reproductive system development and function were underexpressed in normozoospermic infertile men (Table 1). These proteins were involved in the fertilization process and binding of the gonadal cell lines (Table 1). Additionally, the proteins related to the ubiquitination pathway were underexpressed in normozoospermic infertile men (Table 2).

### 3.4. Protein Networks in Normozoospermic Infertile Men

**Figure 4:**
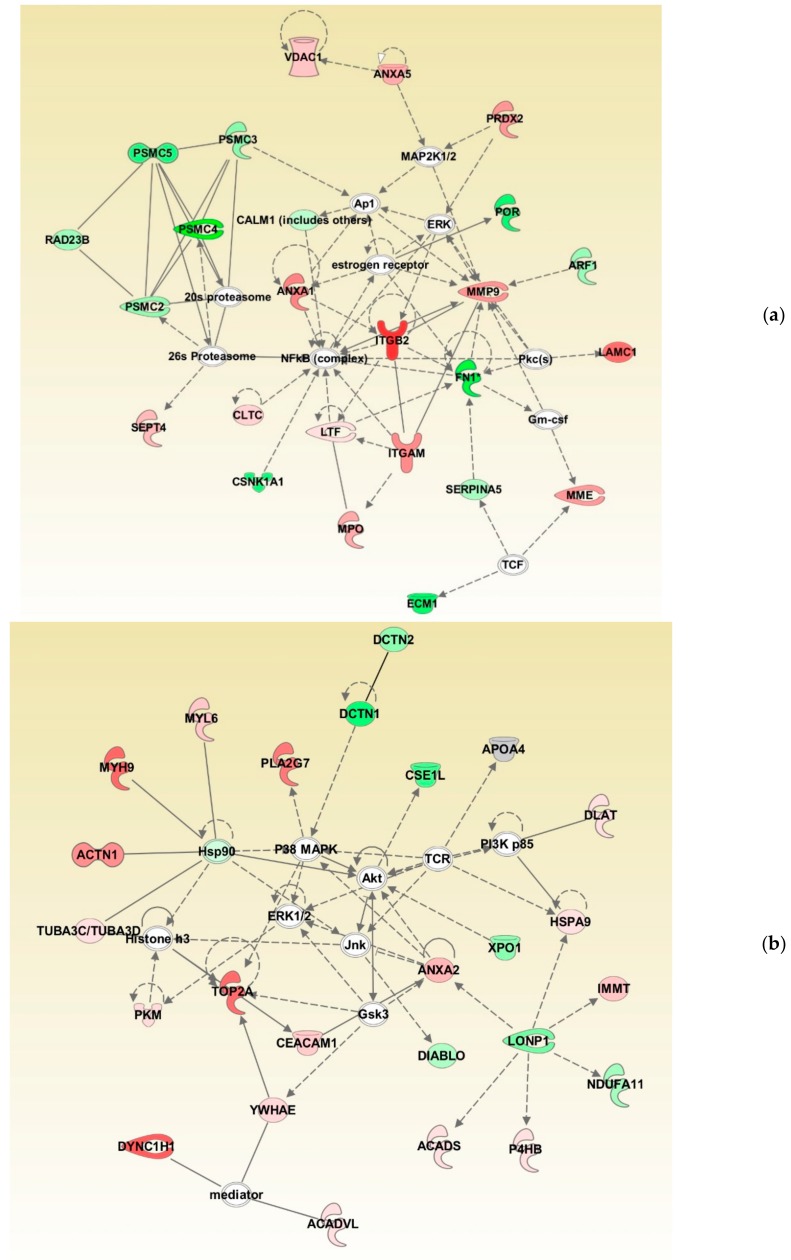
Differentially expressed proteins involved in top networks: (**a**) cellular compromise, inflammatory response, cell-to-cell signaling, and interaction; (**b**) organismal injury and abnormalities, cell death, and survival. These non-directional protein networks were generated using IPA.

Bioinformatic analysis identified the involvement of maximum DEPs in cellular compromise, the inflammatory response, cell-to-cell signaling, and interaction. The network also revealed the interaction between the proteins involved in the ubiquitination pathway with 20 s proteasome and 26 s proteasome complexes (Figure 4a). Furthermore, network analysis revealed ANXA2 as the focus molecule associated with organismal injury and abnormalities, cell death, and survival (Figure 4b).

### 3.5. Western Blot Analysis of Validated DEPs

**Figure 5:**
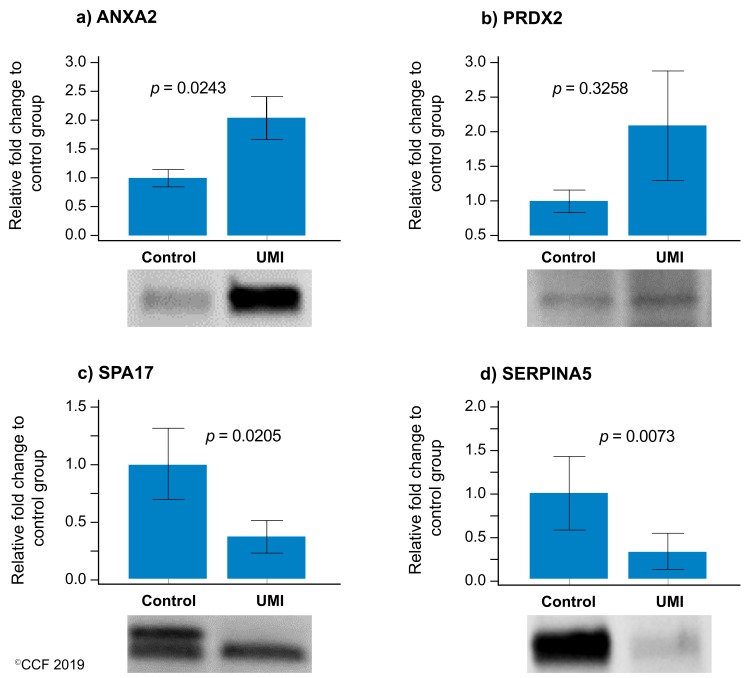
Protein expression levels of the differentially expressed proteins selected for validation by Western blotting in UMI: normozoospermic infertile men (n = 10) relative to control: normozoospermic fertile men (n = 10). (**a**) ANXA2 (**b**) PRDX2, (**c**) SPA17, and (**d**) SERPINA5. Results are expressed as mean ±SEM and in fold variation to normozoospermic fertile men. UMI: unexplained male infertility.

Of the four validated proteins, SPA17 and SERPINA5 were underexpressed and ANXA2 was overexpressed (*p* < 0.05) in UMI subjects (Figure 5), whereas the expression level of PRDX2 was comparable for normozoospermic fertile and infertile men (Figure 5).

## 4. Discussion

Extensive research is being carried out on spermatozoon using the omics platform, mainly to understand the underlying etiologies of male infertility at a molecular level. Normozoospermic infertile men with unexplained male infertility is one such condition that requires high throughput proteomic techniques to decipher the hidden factors affecting the fertility in these men. To date, seven proteomic studies have highlighted the mechanisms/pathways altered in the spermatozoa of normozoospermic infertile men [8,10,11,12,13,18,19]. Different proteomic approaches were used to demonstrate the role of proteins associated with vital sperm functions such as motility, capacitation, and binding of sperm–oocyte vital for successful fertilization. However, each study had its own limitations (Table 3). In the current study, we used the LC-MS/MS platform to profile the maximum number of sperm proteins. Furthermore, the proteins associated with the reproductive function and fertilization process were validated using the WB technique.

Oxidative stress is reported as a cause of infertility in the UMI condition [4,20] and affects the function of the spermatozoa. Sperm protein expression levels are reported to be altered during a continuous state of oxidative stress (reviewed by Agarwal et al. 2014) [21]. In the current study, canonical pathways such as cellular compromise, cell death and survival, and free radical scavenging were highly enriched in UMI subjects (Figure 2). Earlier, we have also demonstrated that energy metabolism and regulation, protein modifications, and oxidative stress-associated proteins are differentially expressed in infertile men with a high number of reactive oxygen species [22]. Furthermore, the proteins associated with the antioxidant mechanism, such as peroxiredoxin, are underexpressed in spermatozoa with oxidative stress [22,23]. In the current study, network analysis demonstrated the interaction between PRDX2 and the extracellular signal-regulated kinases (ERK) pathway (Figure 4a). Spermatozoa generates low levels of physiological ROS required to trigger capacitation and fertilization events. This process is mediated by the ERK pathway [24]. In the current study, the overexpression of the PRDX2 protein suggests a state of oxidative stress and dysregulation of the ERK pathway affecting the function of the spermatozoa in UMI patients. The overexpression of antioxidant protein PRDX2 depicts a state of oxidative stress which may have an adverse effect on the fertilizing ability of the spermatozoa. However, validation of PRDX2 using the Western blot technique revealed that the protein levels were comparable in both the normozoospermic fertile and normozoospermic infertile men.

Global proteomic results revealed the downregulation of ubiquitination pathway proteins in normozoospermic infertile men (Figure 4a). In general, the ubiquitination process regulates the sperm function and morphogenesis [25]. The 26S proteasome complex associated with the ubiquitination process mediates the capacitation of spermatozoa [26]. In the current study, bioinformatic analysis revealed that the proteasome complex proteins PSMC2, PSMC3, PSMC4, and PSMC5 interact with the 20s proteasome and 26s proteasome proteins in the network (Figure 4a). Furthermore, the underexpression of proteasome system proteins (PSMC2, PSMC3, PSMC4, and PSMC5) in men with UMI indicates compromised sperm capacitation as an effect of defective spermatogenesis. It is also reported that the proteasomal proteins are involved in the sperm–zona binding of the capacitated spermatozoa [26,27]. In order to understand the binding of spermatozoa, we have validated the expression of ANXA2 using the WB technique. Annexins are calcium-dependent membrane binding proteins and specifically, ANXA2 is involved in sperm binding to the oviduct of the female reproductive tract, which is essential for the fertilization process [28]. In addition, ANXA2 is associated with the ubiquitination process [29] and its expression is higher during abnormal ubiquitination [30]. Our proteomic results in par with the Western blot results indicate that the overexpression of ANXA2 and underexpression of proteasomal proteins in the spermatozoa may lead to an impaired sperm function in normozoospermic infertile men.

Sperm proteins directly related to the reproductive function play a critical role in the fertilization process. Studies with normozoospermic infertile men reported the underexpression of protein involved in the fertilization process (reviewed by Bracke et al., 2018) [9]. Lui et al., 2018 validated the acrosin-binding protein (ACRBP) and zona pellucida-binding protein 1 (ZPBP1) associated with the fertilization process in in vitro fertilization failure patients [18]. In the current study, A-kinase anchoring protein 3 (AKAP3), AKAP4, phospholipase A2 Group VII (PLA2G7), and SPA17 were identified as being involved in the fertilization processes (Table 1). AKAP proteins are essential for sperm motility. AKAP4 binds with AKAP3 and they are then localized in sperm flagellum [31]. The degradation of AKAP3 and subsequent tyrosine-dephosphorylation results in the capacitation of spermatozoa [32]. Overexpression of AKAP3 in normozoospermic infertile men suggests the accumulation of AKAP3, thus hampering the sperm capacitation process in UMI patients. The other protein, SPA 17, is a mannose-binding protein and is involved in acrosome reactions and the fertilization process [33]. In our earlier study, we reported the underexpression of SPA17 in men with primary or secondary infertility [34]. Therefore, underexpression of SPA17 may affect sperm capacitation and sperm–oocyte binding in normozoospermic infertile patients. The N-terminus of SPA17 shares a similar sequence with cAMP-dependent protein kinase A regulatory subunit II (PKA RII), which has an important role in sperm motility. Its C-terminus plays a likely role in cell–cell adhesion. In general, the C-terminal calmodulin domain of SPA17 (20 KDa) is proteolytically cleaved to 17 KDa after the acrosome reaction and binds to the extracellular matrix of the oocyte [35,36]. WB results of SPA17 revealed two bands in normozoospermic fertile men and one band in normozoospermic infertile men, indicating a compromised acrosome reaction in men with UMI. Validation of SPA17 by WB is in concordance with the proteomic findings, suggesting that SPA17 may be a potential biomarker for screening the men with UMI.

Proteins present in the head of the spermatozoa play a vital role in the fertilization process and are involved in zona–pellucida binding [37]. SERPINA5 was identified as the core protein present in the head of the spermatozoa [38]. Deficiency of SERPINA5 results in a defective spermatogenesis process and is a cause of infertility [39,40]. The expression of SERPINA5 is correlated with the morphology of the spermatozoa [39] and sperm–egg binding mechanism [41,42]. In the current study, IPA analysis identified SERPINA5 interacting with FN1, which is reported to be associated with the capacitation of spermatozoa (Figure 4a). Underexpression of FN1 and SERPINA5 indicates capacitation failure followed by a defective fertilization process in the UMI patients. Therefore, SERPINA5 protein underexpression in normozoospermic infertile men compared to fertile men provides an insight into its role in infertile men with normal semen parameters. Furthermore, validation of SERPINA5 in spermatozoa signifies this protein as a candidate marker for identifying the cause of infertility in normozoospermic infertile men.

The comparative proteomic analysis of sperm proteome and WB analysis revealed that the proteins associated with the fertilization process are dysfunctional in normozoospermic infertile men. In the present study, we limited our WB validation to four proteins (PRDX2, ANXA2, SPA17, and SERPINA5) associated with sperm function. However, validation of the additional DEPs is required to decipher the role of other sperm proteins in the molecular pathology of UMI subjects. In addition, we did not use unique peptides for protein identification and quantification.

## 5. Conclusions

Global proteomic analysis identified altered reproductive pathways in the spermatozoa of normozoospermic infertile men. Validation of key DEPs signifies the importance of ANXA2, SPA17, and SERPINA5 as candidate biomarkers for screening the fertilization potential of spermatozoa in UMI subjects.

## Figures and Tables

**Table 1:** Upregulated proteins associated with reproductive system development and function in normozoospermic infertile men.

Functions	*p*-Value	Upregulated Proteins	Downregulated Proteins
Fertilization	0.0000147	AKAP3, AKAP4, PLA2G7	SPA17
Binding of gonadal cell lines	0.0000148	ANXA5, ITGAM, ITGB2, LTF	FN1

ITGAM: Integrin α-M, ITGB2: Integrin β-2, LTF: Lactotransferrin.

**Table 2:** Downregulated proteins related to the ubiquitination pathway in normozoospermic infertile men.

Symbol	Protein Name	Expr Fold Change	*p*-Value
HSP90B1	heat shock protein 90 β family member 1	−0.664	0.000359
PSMC2	26S protease regulatory subunit 7 isoform 1	−1.693	0.00723
PSMC3	26S protease regulatory subunit 6A	−1.737	0.00195
PSMC4	26S protease regulatory subunit 6B isoform 1	−4.916	0.000978
PSMC5	26S protease regulatory subunit 8 isoform 1	−2.587	0.00319
UCHL1	ubiquitin carboxyl-terminal hydrolase isozyme L1	−1.698	0.00524

**Table 3:** Sperm proteomic studies in normozoospermic infertile men or unexplained infertility.

Study/Authors	Study Description	Proteomic Technique	Western Blot Validation	Limitations
Pixton et al., 2004 [12]	• Fertilization failure (n = 1) • Fertile donors (n = 3)	• 2DE and MS/MS • Pooled sample	• Not performed	• Less sample size • Lack of WB validation
Xu et al., 2012 [8]	• Fertile men (n = 10) • Infertile men (n = 10)	• 2-DE and MALDI-TOF/TOF • Pooled sample	• Proteins: PAEP, ODFP, SEMG1, PSA, GPx4Pre • Individual samples (n = 10)	• Lacks the WB validation of proteins associated with fertilization process, except for ODFP
Frapsauce et al., 2014 [13]	• Fertilization failure with IVF (n = 3) • Successful fertilization with IVF (n = 3)	• 2D-DIGE and MS • Pooled sample	• Not performed	• Use of DIGE • Lack of WB validation
McReynolds et al., 2014 [19]	• Infertile men (n = 12)	• LC-MS/MS • Pooled sample	• Protein: CLU • Individual samples (n = 12)	• WB validation of only one protein CLU
Azpiazu et al., 2014 [10]	• No pregnancy with IVF (n = 15) • Pregnancy with IVF (n = 16)	• LC-MS/MS • Pooled sample (n = 10)	• Protein: SRPK1 • Individual samples ○ No pregnancy (n = 6) ○ Pregnancy (n = 7)	• WB validation of only one protein SRPK1
Légaré et a., 2014 [11]	• Fertile (n = 3+ n = 3) • Infertile (n = 6) • IVF failure (n = 4)	• iTRAQ and LC-MS/MS • Two pools of fertile group • Single pool of infertile and IVF failure group	• Proteins: SEMG1, PIP, GAPDHS, PGK2 • Individual samples • Fertile (n = 13) • IVF failure (n = 14)	• Lacks the WB validation of proteins associated with fertilization process
Liu et al., 2018 [18]	• Pregnancy with IVF (n = 20) • Pregnancy with ICSI (n = 20)	• iTRAQ and LC-MS/MS • Pooled sample	• Proteins: ZPBP1, ACRBP • Individual samples (n = 12)	• Lacks the comparison with control group (fertile men)

## Supplementary Materials

The following are available online at https://www.mdpi.com/2073-4425/10/7/522/s1: Table S1: List of primary and secondary antibodies, Table S2: Semen parameters in normozoospermic fertile men and normozoospermic infertile men, Table S3: (a) Semen parameters in normozoospermic fertile men (n = 18), (b) Semen parameters in normozoospermic infertile men (n = 19) and Table S4: Differentially expressed proteins their abundance in normozoospermic infertile men (UMI cases) compared with normozoospermic fertile men.
