# Supplementary material for: Sperm Proteome Analysis and Identification of Fertility-Associated Biomarkers in Unexplained Male Infertility
Source: Genes (Basel). 2019 Jul 11;10(7):522. doi: 10.3390/genes10070522 (PMC6678187; doi:10.3390/genes10070522)
Supplement: Supplementary file 1 [file genes-10-00522-s001.zip › Supplementary tables/Supplementary Table 1.docx]

| **Primary** | | | | | **Secondary** | | | |
| --- | --- | --- | --- | --- | --- | --- | --- | --- |
| **Protein** | **Antibody** | **Source** | **Manufacturer** | **Dilution** | **Antibody** | **Source** | **Manufacturer** | **Dilution** |
| SPA17 | Anti-Human Rabbit IgG | Rabbit polyclonal | ab172626 | 1:1000 | Anti-Rabbit  Goat IgG | Goat polyclonal | ab97051 | 1:10000 |
| PRDX2 |  |  | ab71533 | 1:1000 |  |  |  |  |
| SERPINA5 | Anti-Human Mouse IgG | Mouse polyclonal | ab172060 | 1:500 | Anti-Mouse  Rabbit IgG | Rabbit polyclonal | ab6728 | 1:10000 |
| ANXA2 |  | Mouse monoclonal | ab54771 | 1:1000 |  |  |  |  |

**Supplementary Table 1:** List of primary and secondary antibodies
