# Supplementary material for: Sperm Proteome Analysis and Identification of Fertility-Associated Biomarkers in Unexplained Male Infertility
Source: Genes (Basel). 2019 Jul 11;10(7):522. doi: 10.3390/genes10070522 (PMC6678187; doi:10.3390/genes10070522)
Supplement: Supplementary file 1 [file genes-10-00522-s001.zip › Supplementary tables/Supplementary Table 2.docx]

**Supplementary Table 2:** Semen parameters in normozoospermic fertile men and normozoospermic infertile men

| **Parameter** | **Fertile men**  **(n=8)** | **Infertile men**  **(n=9)** | **P value** |
| --- | --- | --- | --- |
| Volume (mL) | 4.60 ± 2.22 | 4.34 ± 1.19 | 0.6296 |
| Sperm concentration (10^6^/mL) | 73.10 ± 17.06 | 64.10 ± 46.79 | 0.3359 |
| Sperm motility (%) | 56.00 ± 12.00 | 59.00 ± 9.00 | 0.2895 |
| Sperm normal morphology (%) | 8.25 ± 3.15 | 7.44 ± 1.81 | 0.6993 |

Statistical analysis was performed between normozoospermic fertile men and normozoospermic infertile men. For all values, P < 0.05 indicate a significant difference based on the Mann-Whitney test. Sperm concentration and motility, values are presented as mean ± SD
