# Supplementary material for: Sperm Proteome Analysis and Identification of Fertility-Associated Biomarkers in Unexplained Male Infertility
Source: Genes (Basel). 2019 Jul 11;10(7):522. doi: 10.3390/genes10070522 (PMC6678187; doi:10.3390/genes10070522)
Supplement: Supplementary file 1 [file genes-10-00522-s001.zip › Supplementary tables/Supplementary Table 3.docx]

**Supplementary Table 3a:** Semen parameters in normozoospermic fertile men (n=18)

| **Samples** | **Volume (mL)** | **Sperm concentration (10^6^/mL)** | **Sperm motility (%)** | **Sperm normal morphology (%)** |
| --- | --- | --- | --- | --- |
| *C1 | 6.5 | 68.5 | 59 | 12 |
| *C2 | 4.2 | 97.6 | 46 | 7 |
| *C3 | 3.9 | 91.4 | 50 | 7 |
| *C4 | 2.6 | 79 | 85 | 9 |
| *C5 | 2.7 | 65.9 | 43 | 5 |
| *C6 | 4.2 | 72.4 | 60 | 9 |
| *C7 | 9.2 | 68 | 49 | 13 |
| *C8 | 3.5 | 42 | 59 | 4 |
| **C9 | 7.0 | 46.4 | 75 | 4 |
| **C10 | 4.5 | 81 | 74 | 8 |
| **C11 | 4.2 | 103 | 56 | 5 |
| **C12 | 2.4 | 42.5 | 82 | 13 |
| **C13 | 6.0 | 115 | 72 | 9 |
| **C14 | 9.2 | 154.2 | 68 | 5 |
| **C15 | 2.7 | 69 | 77 | 4 |
| **C16 | 3.5 | 113 | 71 | 5 |
| **C17 | 5.1 | 103 | 56 | 7 |
| **C18 | 5.3 | 76.00 | 71 | 4 |

*Samples used for proteomic analysis. **Samples used for western blot analysis. C= control group (normozoospermic fertile men).

**Supplementary Table 3b:** Semen parameters in normozoospermic infertile men (n=19)

| **Samples** | **Volume (mL)** | **Sperm concentration (10^6^/mL)** | **Sperm motility (%)** | **Sperm normal morphology (%)** |
| --- | --- | --- | --- | --- |
| *P1 | 4.0 | 25.6 | 54 | 7 |
| *P2 | 2.9 | 24.4 | 45 | 6 |
| *P3 | 4.5 | 38.3 | 60 | 5 |
| *P4 | 5.0 | 49.6 | 67 | 9 |
| *P5 | 4.2 | 25.6 | 52 | 8 |
| *P6 | 3.6 | 150.7 | 60 | 8 |
| *P7 | 4.2 | 98.4 | 62 | 10 |
| *P8 | 7.1 | 44.9 | 74 | 9 |
| *P9 | 3.6 | 119.4 | 54 | 5 |
| **P10 | 1.6 | 83.5 | 69 | 4 |
| **P11 | 3.1 | 57.6 | 44 | 6 |
| **P12 | 5.0 | 62.3 | 59 | 6 |
| **P13 | 3.9 | 73.1 | 49 | 6 |
| **P14 | 3.1 | 43.4 | 59 | 6 |
| **P15 | 1.6 | 83.5 | 69 | 4 |
| **P16 | 8.5 | 72.1 | 47 | 4 |
| **P17 | 4.2 | 25.6 | 52 | 8 |
| **P18 | 3.8 | 177 | 70 | 8 |
| **P19 | 2.9 | 47.1 | 61 | 4 |

*Samples used for proteomic analysis. **Samples used for western blot analysis. P= patient group (normozoospermic infertile men).
